# The Immediate Reduction of the Angular Deformity of Spinal Caries

**Published:** 1897-10

**Authors:** 


### THE IMMEDIATE REDUCTION OF THE ANGULAR DEFORMITY OF SPINAL CARIES.

The familiar sight of angular deformity of the spine as a result of caries has probably tended to blind us to the possibility of lessening the disfigurement, and has led physicians to accept this result as satisfactory, and to assure patients that nothing could be done to improve the condition. This advice is backed by the authority of the combined writers and teachers of all ages down to the present time. It is little wonder, therefore, when Calot described some months ago his method for the immediate, forcible reduction of angular deformities of the spine that the announcement should excite almost no attention.

The matter has been taken up, however, in England by Robert Jones and A. H. Tubby (*British Medical Journal*, August 7, 1897), and their experience so fully corroborates that reported by Calot that it seems proper to make this preliminary announcement. Although Jones and Tubby profess to having regarded the accounts published by Calot, of Berek-sur-Mer, and Redard, of Paris, in a somewhat skeptical spirit, as no doubt most other readers also did, and being further influenced by the authoritative statements contained in all the text-books that the only safe way of treating carious disease of the spine is by a long course of rest, and if deformity followed, as it very often does, in must be accepted as a lamentable but unavoidable sequence of the disease; still, they decided to put the question to a cautious trial.

The possibility of causing paresis or even paralysis, either temporary or permanent, by forcible straightening of the spine was not lost sight of, and the danger of lighting up a fresh inflammatory process or of inducing an acute general dissemination of the tubercular process were well considered.

The writers have followed the method of Calot; they prepared themselves by personal observations in the French clinics. The procedure is as follows: The patient is fully etherized and placed in the prone position; assistants then make firm, steady traction, simultaneously upon the head, arms, and legs for a few minutes. The surgeon then makes downward pressure with his hands upon the prominence, counter-pressure being afforded by the open palms of the assistant placed upon the abdomen. It is advised that the patient's bowels be well emptied before the operation, as the assistants' hands can then readily support the anterior aspect of the spine, and so prevent a too rapid reduction of the deformity. If the projection is dorsal, counter-pressure may be made indirectly through the chest walls. The patient must be kept under an anæsthetic throughout. After reduction, a plaster of Paris corset is applied, the head and neck being included, leaving exposed the face only. During the application of the jacket, the spine must be maintained in hyperextension, and pressure kept up on the site of the previous deformity. The most convenient way to apply the corset is by first suspending the patient by means of a Sayre's apparatus.

Owing to the difficulty of not being always able to obtain a sufficient number of assistants, Jones and Tubby have devised a special apparatus for making traction during the manipulations of straightening the spine.

The authors report their experience with eleven cases. In six, immediate and complete reduction was obtained; two of these, in both of which the deformity was of an extreme type, were remarkable on account of the facility with which the reduction was effected. In five cases partial reduction only was obtained; in three of these the angle was very sharp, and the disease limited to two or three vertebræ. In the other two the projection was sharp, but obtuse, and extended over six or seven vertebræ, and was accompanied by much thickening of the tissues at the site of the disease. Of the five cases of partial reduction were the first two operated upon, and on account of lack of any experience to serve as a guide, less force was used than was subseqnently found to be necessary to completely remove the deformity. In the other three cases the consolidation of the spine was such as to resist a large amount of force; still, in all of them, although the reduction was incomplete, there was considerable lessening of the projection (estimated) at about three-fourths), and an increase in the length of the spinal column of an inch or more.

In not a single case has paresis or paralysis followed the operation, and in four cases observed for two months no untoward symptoms of any kind has appeared.

The authors conclude that, although the method is still on trial as to its ultimate results, it seems to afford a good prospect of reducing the deformity without incurring dangerous consequences. They promise a more detailed account in the near future.

Calot has gone a step further, having cut down on the spinous processes in some cases, and, after reduction, wired them together to maintain extension of the column.

Such, then, is the present status of this new procedure. If we accept the reports, is we are bound to, we must conclude that a valuable contribution has been made to the treatment of spinal caries; a treatment not appropriate until inflammatory symptoms have subsided; not applicable to all cases, and one that requires the exercise of good judgment in its execution, but still highly useful in many instances.

In this matter, as in medicine generally, the best results will follow a keen discrimination in the selection of cases, and an intelligent application of the appropriate measures.—*University Medical Magazine.*

